# Learning meaningful latent space representations for patient risk stratification: Model development and validation for dengue and other acute febrile illness

**DOI:** 10.3389/fdgth.2023.1057467

**Published:** 2023-02-22

**Authors:** Bernard Hernandez, Oliver Stiff, Damien K. Ming, Chanh Ho Quang, Vuong Nguyen Lam, Tuan Nguyen Minh, Chau Nguyen Van Vinh, Nguyet Nguyen Minh, Huy Nguyen Quang, Lam Phung Khanh, Tam Dong Thi Hoai, Trung Dinh The, Trieu Huynh Trung, Bridget Wills, Cameron P. Simmons, Alison H. Holmes, Sophie Yacoub, Pantelis Georgiou, on behalf of the Vietnam ICU Translational Applications Laboratory (VITAL) investigators

**Affiliations:** ^1^ Centre for Bio-Inspired Technology, Imperial College London, London, United Kingdom; ^2^ Centre for Amtimicrobial Optimisation, Imperial College London, London, United Kingdom; ^3^ NIHR HPRU in Healthcare Associated Infections and Antimicrobial Resistance, Imperial College London, London, United Kingdom; ^4^ Oxford University Clinical Research Unit, Ho Chi Minh City, Vietnam; ^5^ Hospital for Tropical Diseases, Ho Chi Minh City, Vietnam; ^6^ University of Medicine and Pharmacy, Ho Chi Minh City, Vietnam; ^7^ Children’s Hospital No 1, Ho Chi Minh City, Vietnam; ^8^ Centre for Tropical Medicine and Global Health, Nuffield Department of Medicine, University of Oxford, Oxford, United Kingdom; ^9^ Institute of Vector Borne Disease, Monash University, Melbourne, VIC, Australia

**Keywords:** autoencoder (AE) neural networks, unsupervised learning, similarity retrieval, visualisation, clinical decision support system (CDSS), dengue

## Abstract

**Background:**

Increased data availability has prompted the creation of clinical decision support systems. These systems utilise clinical information to enhance health care provision, both to predict the likelihood of specific clinical outcomes or evaluate the risk of further complications. However, their adoption remains low due to concerns regarding the quality of recommendations, and a lack of clarity on how results are best obtained and presented.

**Methods:**

We used autoencoders capable of reducing the dimensionality of complex datasets in order to produce a 2D representation denoted as latent space to support understanding of complex clinical data. In this output, meaningful representations of individual patient profiles are spatially mapped in an unsupervised manner according to their input clinical parameters. This technique was then applied to a large real-world clinical dataset of over 12,000 patients with an illness compatible with dengue infection in Ho Chi Minh City, Vietnam between 1999 and 2021. Dengue is a systemic viral disease which exerts significant health and economic burden worldwide, and up to 5% of hospitalised patients develop life-threatening complications.

**Results:**

The latent space produced by the selected autoencoder aligns with established clinical characteristics exhibited by patients with dengue infection, as well as features of disease progression. Similar clinical phenotypes are represented close to each other in the latent space and clustered according to outcomes broadly described by the World Health Organisation dengue guidelines. Balancing distance metrics and density metrics produced results covering most of the latent space, and improved visualisation whilst preserving utility, with similar patients grouped closer together. In this case, this balance is achieved by using the sigmoid activation function and one hidden layer with three neurons, in addition to the latent dimension layer, which produces the output (Pearson, 0.840; Spearman, 0.830; Procrustes, 0.301; GMM 0.321).

**Conclusion:**

This study demonstrates that when adequately configured, autoencoders can produce two-dimensional representations of a complex dataset that conserve the distance relationship between points. The output visualisation groups patients with clinically relevant features closely together and inherently supports user interpretability. Work is underway to incorporate these findings into an electronic clinical decision support system to guide individual patient management.

## Introduction

The adoption of electronic health records (EHRs) in routine clinical practice has led to an increase in the availability and quality of medical data in an electronic format. In addition, large datasets obtained from healthcare delivery or as the result of research studies contribute to an increasingly large knowledge base. Data availability allows for development of some advanced clinical decision support systems (CDSSs) which aid clinicians in making faster, better informed, and cost-effective decisions at the point of use (1): these systems can provide clinicians with electronic alerts or reminders, patient-specific diagnostics, and automated treatment recommendations (2–5). Specific tools have also been designed to predict the likelihood of infection (6, 7), automate drug dosing and prescriptions (8, 9), and evaluate a patient’s risk of complications (10–12) or even death (13).

Whilst some CDSSs have demonstrated the ability to improve the quality of clinical care (14, 15), overall adoption remains low partly due to inherent issues with EHRs and the automation of healthcare decisions (16, 17). EHR platforms and CDSSs are typically implemented independently, leading to incompatibilities and inconsistencies in how data is recorded (18). Furthermore, the black-box approaches taken by some systems have induced concern from clinicians regarding the quality and interpretability of such recommendations (19, 20). Clinicians typically rely on existing knowledge when making decisions and consider previous patients when diagnosing or formulating a treatment plan (21, 22)—therefore clarity on how CDSS recommendations are produced and conveyed to the clinician are essential aspects which affect utility and uptake (23).

To circumvent these challenges, the use of dimensionality reduction techniques often coupled with visualisation strategies have been widely used. These techniques transform the original high-dimensional space into a low-dimensional representation which retains the most relevant properties of the original data (24) and can be used for noise reduction, data visualization or as an intermediate step to facilitate other analyses. The transformations are commonly divided into linear and non-linear approaches. A well-known linear approach is Principal Component Analysis (PCA) which has been widely used to visualise health care data (25). Similarly, t-distributed Stochastic Neighbor Embedding (t-SNE) and Uniform Manifold Approximation and Projection (UMAP) have been proposed as non-linear alternatives focusing on visualisation (26, 27). In the last years, given the success achieved by deep learning models, the use of Autoencoders to extract information from complex electronic health care data has grown considerably (28–30). While some studies have demonstrated that these representations can capture relevant clinical insights from raw data, the results and visualisations produced are often ineffective for use in routine clinical practice.

This study proposes a methodology which relies on unsupervised techniques of reducing data complexity in a meaningful way such that complex information can be relayed to the end user through accessible and comprehensible graphical representations. In this manuscript, autoencoders, a type of neural network, has been employed on a real-world dataset of patients with dengue and acute febrile illness to serve as an exemplar to demonstrate its role and utility.

### Clinical picture of dengue infections

Dengue is a systemic viral disease which exerts a significant health and economic burden worldwide. There are an estimated 51 million symptomatic cases each year, with seasonal epidemics and high caseloads imposing a huge strain on local healthcare services (31). The wide spectrum, and non-specific nature of clinical presentations pose further challenges to effective healthcare planning (32).

The course of infection typically exhibits three distinct phases: febrile, critical, and recovery (33, 34). The febrile phase involves high fever and is associated with generalised muscle pain lasting around two to seven days (34). Nausea, vomiting and abdominal pain may also occur (35). In a small proportion, the disease proceeds unpredictably to a critical phase associated with resolution of fever with an increase in blood haematocrit and a decrease in platelet levels (34). During this period, the leakage of plasma from the blood vessels may result in fluid accumulation in sites including the chest and abdominal cavity (34). There may also be associated organ dysfunction and severe bleeding such as from the skin or gastrointestinal tract (36). Shock (dengue shock syndrome) and haemorrhage occur in less than 5% of all cases of dengue; (36) however, those who have previously been infected with other serotypes of dengue virus (secondary infection) are at an increased risk (37). These outcomes occur relatively more commonly in children and young adults (34, 37). This is usually followed by the recovery phase with resorption of the leaked fluid into the bloodstream and resolution of illness.

Strategies to identify patients at increased risk of complications such as dengue shock syndrome (DSS) during the early febrile phase of illness are important priorities to improving healthcare organisation and delivery (38, 39). A widely adopted approach appropriate in low- and middle-income countries (LMICs) is the use of clinical warning signs outlined in the World Health Organisation (WHO) 2009 dengue guidelines (33). These guidelines have relatively few requirements for implementation, needing only clinical examination findings and results from basic haematological tests. While absence of these signs provides a high negative predictive value for severe dengue (40), real world findings using these systems have shown variability in performance (41), and the systems have not resulted in lower rates of potentially unnecessary admissions (42).

## Materials and methods

**Figure 1:**
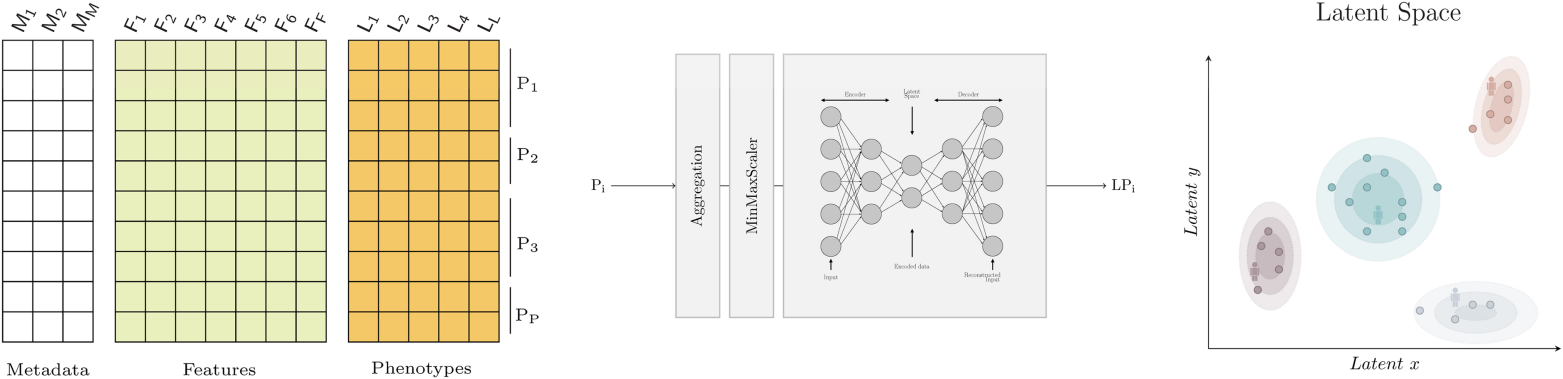
Graphical abstract. On the left, the dataset with metadata, features and phenotypes where each row represents a daily patient profile. In the middle, the model that transforms a patient stay with one or more daily profiles (P_*i*_) into a two dimensional embedding (LP_*i*_) for visualisation. The aggregation step is used to describe the worst patient status using the aggregation functions shown in Table 1. The embeddings are obtained using autoencoders. On the right, the latent space where similar patients are grouped together. Each point represents a patient and the shaded areas represent the density distribution; that is, the concentration of patients for which the phenotype of interest occurs. Note that the latent space can be used to visualise any feature or phenotype of interest.

**Figure 2:**
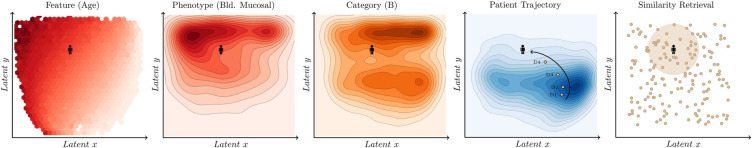
Latent space analysis. From left to right, the latent space produced can be described in terms of features using the average value (e.g. age) and phenotypes (e.g. mucosal bleeding) or categories (e.g. category which is associated with the warning signs defined in the WHO 2009 dengue guidelines) using the density distribution. In addition, it is possible to visualise the evolution of the patient over time (patient trajectory) and retrieve previous past similar patients to support decision making (similarity retrieval).

Learning a meaningful representation of high dimensional data in two dimensions (latent space) has two potential benefits. First, it enables a better, more intuitive understanding of an otherwise complex dataset to clinician end-users through visualisations (see Figure 1). As an example, the latent space produced can be thoroughly described in terms of training features, phenotypes of interest, categories used for patient stratification, or individual patient trajectories over time. Second, it facilitates the projection of unseen observations into the latent space which can be used for efficient similarity retrieval (see Figure 2).

### Dataset

The dataset used in the study consists of an aggregation of prospective clinical data conducted at the Hospital of Tropical Diseases (HTD) and collaborator hospitals in Ho Chi Minh City, Vietnam by Oxford University Clinical Research Unit (OUCRU) between 1999 and 2021. The studies were carried out in both outpatient and inpatient settings with varying patient populations (43–45). Only children (under 18 years old) have been considered, since they were the most commonly represented in the datasets and there are separate paediatric and adult dengue guidelines.

The included data is derived from 12,884 patients and their 19516 complete daily profiles (where all input features were available) attending a healthcare facility with an acute febrile illness compatible with dengue. Overall, 4,344 (33.7%) of the patients in the dataset were ultimately diagnosed with dengue infection through laboratory investigations. Dengue diagnosis was defined as one of: (i) a positive NS1 point of care assay or NS1 ELISA, (ii) positive reverse transcriptase polymerase chain reaction (RT-PCR), (iii) positive dengue IgM through acute serology, (iv) or seroconversion of paired IgM samples where available. A complementary dataset with the “worst patient status” during the study period has been created using the aggregation functions defined in Table 1. Exclusively patients with all the selected features available were included in the analysis.

**Table 1:** Input features.

Name	Unit	NRR	Aggregation
Age	year	—	First
Weight	kilograms	—	Mean
Body Temperature	celsius	36.1–37.8	Max
Platelets	k/μL	150–450	Min
Haematocrit	%	36–50	Max

NRR, normal reference range; k/μL, kilocounts per microliter.

### Selected features, phenotypes and categories

After reviewing the scientific literature and discussion with dengue infectious disease experts, five features were selected (see Table 1) meeting the following criteria: (i) routinely available at early stages of the disease, (ii) collected regularly over the assessment period and (iii) deemed to provide appropriate information for patient evaluation.

The three main categories for patient risk stratification are: (i) *Category A* where vascular leakage, significant bleeding, organ impairment or shock occurs, (ii) *Category B* which includes some of the clinical warning signs outlined in the WHO 2009 dengue guidelines (33) and (iii) *Category C* for those patients not included in the two previous categories. Note that categories *A* and *B* are not mutually exclusive; that is, a patient might be in both. On the contrary, category *C* is mutually exclusive with categories *A* and *B*. A detailed description of the previously mentioned compound phenotypes and risk stratification categories is included in Table 2.

**Table 2:** Compound phenotypes and categories.

Name	Phenotypes
Fluid accumulation	Ascites, pulmonary oedema and/or pleural effusion
Vascular leakage	Ascites, pulmonary oedema, respiratory distress and/or pleural effusion
Significant bleeding	Considerable mucosal (e.g. gastrointestinal) bleeding
Organ impairment	Abnormality in central nervous system (CNS) and/or liver (AST>1000 or ALT>1000)
Category A	Any vascular leakage, significant bleeding, organ impairment and/or shock
Category B	Abdominal pain or tenderness, persistent vomiting, ascites, pleural effusion, bleeding mucosal, lethargy, restlessness and/or liver enlargement over 2 cm
Category C	No complications or warning signs

CNS, central neural system; AST, aspartate aminotransferase (U/L); ALT, alanine transaminase (U/L).

### Selected dimensionality reduction algorithm

During the algorithm selection process we have considered linear algorithms such as Principal Component Analysis (PCA), non linear algorithms from the manifold family such as T-distributed Stochastic Neighbor Embedding (t-SNE) and Uniform Manifold Approximation and Projection (UMAP) and neural network based algorithms such as Self-Organising Maps (SOM) and Autoencoders. For the proposed methodology, the algorithms selected must meet the following requirements: (i) are unsupervised and therefore do not require ground truth labels, (ii) the latent space produced is continuous and (iii) unseen samples can be projected on the latent space. The latter two points are particularly important to enable similarity retrieval (see Table 3). In addition to these requirements, autoencoders were selected for the versatility in configuration and data formats and the approach to reduce the dimensionality by encoding-decoding the data and measuring the loss.

**Table 3:** Overview of dimensionality reduction algorithms.

Algorithm	Unsupervised	Continuous space	Unseen data	Comments
PCA	✓	✓	✓	The performance is likely to decrease as dimensionality increases due to its linear nature.
t-SNE	✓	✓		Its inability to reduce the dimensionality of unseen data points makes a real-time similarity retrieval system impossible.
UMAP	✓	✓		It allows to reduce the dimensionality of unseen data points yet the performance is considerably worse than other transfomers.
SOM	✓		✓	The limitations imposed by the discrete space limits the applicability to similarity retrieval.
Autoencoders	✓	✓	✓	The ability to encode unseen samples and its support for higher-dimensional data, including also time-series or images, make it really flexible and ideal for similarity retrieval.

PCA, principal component analysis; t-SNE, T-distributed stochastic neighbor embedding; UMAP, uniform manifold approximation and projection; SOM, self-organising map.

Autoencoders are a type of neural network which aim to learn an encoding of the input data (46). They do so by attempting to copy their input to their output, having gone through a hidden layer h which has fewer neurons than the input has features. This hidden layer h, often called a bottleneck, forces the model to extract the essential features present in the input data to then be able to reconstruct the input as faithfully as possible. Basic autoencoders are composed of two main elements, an encoder and a decoder, as seen in Figure 1. The encoder is used to map the input data to a code or encoding, often called the latent representation. The decoder uses this code to produce a reconstruction of the input. This structure makes autoencoders ideal for dimensionality reduction, as the latent representation will contain only the most important features of the input data. An autoencoder with two neurons in its bottleneck can then be used to visualise data in two dimensions.

### Evaluating performance for model selection

Dimensionality reduction methods extract the meaningful properties of a dataset and, in the process, lose some of the information. Over 1700 hyperparemeter configurations were explored using grid search and their performance evaluated using both distance and density metrics (see Table 4). In addition, the latent space of the best performing model was further analysed to verify its alignment with established characteristics of the dengue disease progression (see Figure 2). Distance metrics can be used to determine how well the distances between points are preserved. The stronger the relationship between the distances in the reduced space and the distances in the high-dimensional space, the less information has been lost. Computing the distances from each point to every other point can be highly computationally intensive. Therefore, sampling points at random from the dataset to be used in the evaluation is sometimes necessary.

**Table 4:** Evaluation metrics.

Type	Metric	Aim	
Distance	Sheppard	Distance preservation	*na*
	Pearson	Distance preservation	↑
	Spearman	Similarity retrieval	↑
	Procrustes	Information loss	↓
Density	Convex hull ratio	Good visualisation	↓
	Concave hull ratio	Good visualisation	↓
	GMM ratio	Good visualisation	↓

GMM, Gaussian mixture model; ↑, higher values are better; ↓, lower values are better.

There are applications where maintaining the ordering of distances is more important than having a linear relationship between the distances in the original and reduced spaces. Similarity retrieval, for example, will provide the same results in the original space and the two-dimensional space if the Spearman rank correlation coefficient of the distances in both spaces is one.

Distance metrics alone are not sufficient when comparing different dimensionality reduction algorithms or models. Indeed, as dimensionality increases, the distance from a point to its nearest neighbour nears the distance to the farthest data point (47). This effect was shown to arise in datasets with as few as ten dimensions (48). As this happens, all points in the dataset will be at a similar distance from one another, which poses issues when using distances between points to assess performance. Similarity retrieval techniques that rely on distance metrics such as Euclidean distance will also become flawed. Therefore, where available, metrics which make use of labels associated with data points should be used in conjunction with distance metrics when evaluating dimensionality reduction algorithms.

#### Sheppard diagram

Sheppard diagrams are scatter plots of two measurements of distances between objects. In dimensionality reduction analysis, the first measurement or collection of distances corresponds to the points in the original dimension. The second measurement is the distances in the reduced space. Plotting one measurement against the other can be used as a visual indication of any distortion incurred when reducing the dimensionality. In other words, it shows how well distances have been preserved relative to one another. Collinear points indicate that there has been no distortion. The more points which do not lie on this line, the more the distances have been distorted and information lost whilst reducing the dimensionality.

#### Pearson’s correlation coefficient

The Pearson correlation coefficient is a statistical measure that gives the linear correlation between two variables. The result is given in the [−1,1] range where 0 indicates no linear correlation (49). A value of 1 indicates that every increase in one variable is accompanied by a rise of fixed proportion in the other. Conversely, a value of −1 indicates that every increase in one variable is accompanied by a decrease of fixed proportion in the other.

#### Spearman’s correlation coefficient

The Spearman’s rank correlation coefficient measures the dependence between the rankings of two variables (50). Values of 1, −1 and 0 respectively indicate a monotonically increasing relationship between the variables, a monotonically decreasing relationship and no relationship.

#### Procrustes analysis

Ordinary or classical Procrustes analysis is a statistical method typically used to compare the shapes of two or more objects. The comparison is achieved by performing Procrustes superimposition, which finds a set of translation, rotation and uniform scaling operations which optimally superimposes the objects (51). An optimal superimposition minimises the Procrustes distance d between objects, typically defined as the square root of the sum of the squared pointwise differences between two objects (see Equation 1) where x and y denote two groups of n points in p dimensions. When comparing points with different dimensionality, the dataset with fewer dimensions should have columns of zeros appended to match the dimension p.

(1) $$d=\sqrt{\sum_{i=1}^{n}\left\{\sum_{\;j=1}^{\;p}(x_{ij}-y_{ij}{)}^{2}\right\}}$$

Procrustes analysis can be used to evaluate the performance of dimensionality reduction algorithms by applying Procrustes superimposition to the original dataset and the data in the reduced space. The final Procrustes distance obtained after the optimal superimposition has been found can be used as a disparity measure between the two sets of points. In this report, the squared disparity is used. A value of zero indicates that the points can be perfectly superimposed and that no information has been lost during the dimensionality reduction process.

#### GMM ratio

The GMM ratio uses Gaussian Mixture Models with one component. The metric takes the ratio of the area of the confidence ellipsoid of a model fitted to data points with a given label to the area of the confidence ellipsoid of a model fitted to all data points. In comparison to the convex hull ratio and the concave hull ratio, this metric is the most robust to outliers as the areas of the ellipsoids are not severely impacted by points that lie far away from the probability distribution’s mean.

### Hyperparameters

Autoencoders have multiple parameters which can impact a model’s performance, including but not limited to the learning rate, batch size, number of layers and layer sizes, and the number of training epochs. In this study, different hyperparameter configurations were explored using grid search and compared using the previously described metrics (see Table 4). In total, 1,728 different configurations were tested using all combinations of hyperparameters shown in Table 5.

**Table 5:** Grid search hyperparameters.

Parameter	Values
Layers${}^{a}$	[]${}^{b}$, [5], [4], [3], [5,4], [5,3], [4,3], [5,4,3], [4,4,3,3]
Activation	“ReLU,” “Sigmoid”
Learning rate	0.005, 0.001, $5\times 10^{-4}$, $1\times 10^{-4}$, $5\times 10^{-5}$, $1\times 10^{-5}$
Epochs	10, 30, 50, 100, 150, 250, 350, 500
Batch size	16, 32

${}^{a}$Layers refers to the hidden layers used in the encoder. The input layer and latent layer are not included. The decoder layers are the mirror image of the encoder layers.

${}^{b}$No hidden layers other than the latent dimension.

### Software

The Python programming language was used in this research. The models and performance metrics from Scikit-learn (52) and PyTorch (53) were employed. Data handling was done with Pandas (54) and data visualisation using Matplotlib (55).

## Results

### Data insights

Demographics and clinical characteristics are described in Table 6 for the overall data and three different categories. The median age was 8 years (IQR, 5–11 years) for all patients in the dataset with 4,344 (33.7%) patients with a subsequent laboratory-confirmed diagnosis of dengue. Patients experiencing complications from dengue in *Category A* tended to be older with a median age of 10 years (IQR, 8–13 years) whereas the gender was distributed evenly across groups, with 7203/12884 [55.9%] male patients. The median maximum haematocrit across groups was 39.8% (IQR, 36.9–44.0%) with higher values seen in *Category A* 45% (IQR, 41–49%). In contrast, the median minimum platelet count was 184 k/μL (IQR, 81–250) with lower values for the *Category A* 68 k/μL (IQR, 36–143).

**Table 6:** Characteristics of patients for the overall worst patient status data and categories A, B and C.

		Missing	Overall	Category A	Category B	Category C
n			12,884	4,866	6,809	4,486
Age${}^{a}$, year		0	8.0 [5.0, 11.0]	10.0 [8.0, 13.0]	8.0 [5.0, 11.0]	6.0 [3.0, 9.0]
Gender	Female	0	5,681 (44.1)	2,180 (44.8)	3,073 (45.1)	1,915 (42.7)
	Male		7,203 (55.9)	2,686 (55.2)	3,736 (54.9)	2,571 (57.3)
Weight, Kg		0	25.0 [18.0, 35.0]	30.0 [24.0, 40.0]	26.0 [19.0, 36.0]	21.0 [15.0, 31.0]
Platelet count${}^{b}$, k/μL		0	184.0 [81.0, 250.0]	68.0 [36.0, 143.0]	139.0 [52.0, 232.0]	225.0 [177.0, 277.0]
Haematocrit, %		0	39.8 [36.9, 44.0]	45.0 [41.0, 49.0]	41.0 [37.7, 46.2]	37.6 [35.4, 39.9]
Body Temperature, celsius		0	37.5 [37.2, 38.0]	37. [37.0, 38.4]	37.5 [37.0, 38.0]	37.4 [37.2, 37.8]
Mucosal bleeding	False	3,162	9,066 (93.3)	1,268 (72.3)	4,016 (86.0)	4,469 (100.0)
	True		656 (6.7)	487 (27.7)	656 (14.0)	
Vomiting	False	2,167	5,333 (49.8)	253 (9.2)	813 (13.1)	4,439 (100.0)
	True		5,384 (50.2)	2,499 (90.8)	5,384 (86.9)	
Abdominal pain	False	2,063	7,773 (71.8)	1,117 (39.9)	2,670 (46.7)	4,486 (100.0)
	True		3,048 (28.2)	1,686 (60.1)	3,048 (53.3)	
Abdominal tenderness	False	283	11,014 (87.4)	3,176 (68.5)	5,114 (78.0)	4,470 (100.0)
	True		1,587 (12.6)	1,459 (31.5)	1,442 (22.0)	
Shock	False	21	10,903 (84.8)	2,891 (59.6)	5,091 (75.0)	4,486 (100.0)
	True		1,960 (15.2)	1,960 (40.4)	1,700 (25.0)	
Vascular leakage	False	0	12,249 (95.1)	4,231 (87.0)	6,222 (91.4)	4,486 (100.0)
	True		635 (4.9)	635 (13.0)	587 (8.6)	
Significant bleeding	False	0	12,756 (99.0)	4,738 (97.4)	6,691 (98.3)	4,486 (100.0)
	True		128 (1.0)	128 (2.6)	118 (1.7)	
Organ impairment	False	0	8,175 (63.5)	157 (3.2)	3,653 (53.6)	4,486 (100.0)
	True		4,709 (36.5)	4,709 (96.8)	3,156 (46.4)	
PCR Dengue serotype	<LOD	2,068	6,748 (62.4)	790 (27.4)	3,099 (54.3)	3,464 (78.1)
	DENV-1		1,957 (18.1)	1,131 (39.3)	1,305 (22.9)	373 (8.4)
	DENV-2		1,066 (9.9)	643 (22.3)	725 (12.7)	206 (4.6)
	DENV-3		321 (3.0)	125 (4.3)	187 (3.3)	99 (2.2)
	DENV-4		706 (6.5)	173 (6.0)	381 (6.7)	297 (6.7)
	Mixed		18 (0.1)	18 (0.7)	12 (0.2)	

sbp, systolic blood pressure; dbp, diastolic blood pressure; GI, gastroinstestinal; CNS, central nervous system; LOD, limit of detection.

Continuous features are provided as median [Q1, Q3] and categorical features as n (%). Categories *A* and B are not mutually exclusive.

${}^{a}$Patients over the age of 18 were filtered out due to the low number of samples in the dataset.

${}^{b}$IQR rule was applied to remove outliers.

Commonly reported symptoms for all patients in the dataset include persistent vomiting (5384/10717 [50.2%]), abdominal pain (3048/10821 [28.8%]) and tenderness (1587/12601 [12.6%]) which consistently appear in patients assigned to *Category A* with 90.8%, 60.1% and 31.5% of patients experiencing these symptoms respectively. These symptoms are part of the standardised warning signs defined in the WHO 2009 dengue guidelines. Among cases with bleeding, this amounted to minor skin bleeding (2715/10334 [26.3%]), mucosal haemorrhage (656/9066 [6.7%]) and significant bleeding (e.g. gastrointestinal) (128/4863 [2.6%]). Within the dataset, (1960/10903 [15.2%]) of patients experienced shock. It is important to note that although all patients included experienced an acute febrile illness, they are subject to specific study inclusion criteria which mean that they are not representative of the overall patient population in Vietnam.

### Model selection

The learning rate dictates the degree to which the neural network’s weights will be updated, with higher values such as 0.1 leading to unstable training, preventing the model from converging and producing satisfactory results. The number of hidden layers in the autoencoder impacts how complex a function it can learn. This directly influences the preservation of distances, with simpler models with fewer layers obtaining distance metric results approaching and exceeding PCA’s (see Table 7). The non-linearity provided by the ReLU function allowed the model to obtain a Pearson coefficient value of 0.940, exceeding the value of 0.916 obtained by PCA. Distance preservation is, however, not the only goal. Points with similar labels should be located near to one another, making similarity retrieval applications more meaningful. Models with more hidden layers produced better density metric results, in particular for the shock label. The added complexity introduced to the model by the layers and activation functions allows it to represent high-dimensional data in 2D better but does it at the expense of distance preservation. However, models with too many hidden layers produced representations with data points very densely grouped in the latent space, impacting visualisation and utility. The goal of the encoder is to reduce the dimension of the data with each new layer. In this case, where only five input features are reduced to two dimensions, introducing new layers has diminishing returns and can negatively impact the model.

**Table 7:** Evaluation metrics for various representative hyperparameter configurations.

Layers	Activation	Pearson	Spearman	Procrustes	GMM	Comments
-	-	0.916	0.896	0.272	0.814	PCA
[ ]	ReLU	0.940	0.920	0.226	0.695	The approximate linearity of the ReLU activation function of this model favours distance preservation
[ ]	Sigmoid	0.917	0.906	0.240	0.543	The non-linearity of the Sigmoid activation affects distance metrics slightly and improves density metrics
[3]	Sigmoid	0.840	0.830	0.301	0.321	It balances distance preservation and density metric results
[5,4,3]	ReLU	0.635	0.622	0.505	0.104	It is a complex model with good density metric results but produces dense points in the latent dimension not apt for visualisation of patient trajectories over time. In addition, distance metric results show that distances are not preserved and therefore it is inadequate for similarity-based retrieval

**Figure 3:**
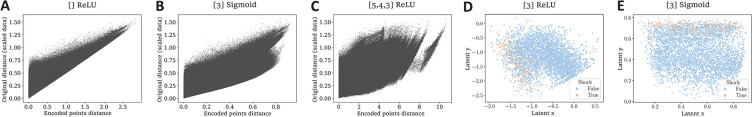
Sheppard diagrams (left) and shock label projections (right). On the left, Sheppard diagrams obtained for autoencoders with A) no hidden layers and ReLU activation, B) one hidden layer with 3 nodes and Sigmoid activation, and C) three hidden layers with 5, 4, and 3 nodes respectively, and ReLU activation. On the right, the distribution of patients in the latent space using an autoencoder with D) one hidden layer with 3 nodes and ReLU activation and E) one hidden layer with 3 nodes and Sigmoid activation. The color indicates whether the patient experienced (orange) or not (blue) an episode of shock during their hospital stay.

Similarly, a network that uses only linear activation functions will produce results similar to those obtained using PCA (56). However, when other activation functions are used, such as ReLU or sigmoid, the model can learn more complex, non-linear mappings. While distance and density metrics are not heavily impacted by the use of ReLU over sigmoid or vice versa, the two-dimensional representation of the points is affected. Using the ReLU activation can cause neurons to be deactivated, producing straight edges in the latent dimension, which can be hard to interpret (see Figure 3D). The sigmoid activation avoids this as it does not entirely deactivate neurons by not producing zero values.

Balancing distance preservation and the density metric results produces results covering more of the latent space, improving visualisation whilst preserving utility, with similar points grouped closer together. In this case, this balance is achieved by only using one hidden layer with three neurons in addition to the latent dimension layer, which produces the output. The Sheppard diagrams in Figures 3A,B,C illustrate differences in the distance preservation achieved by different models.

### The latent space

The latent space is a representation of compressed data in which similar data points are closer together in space and was initially created using data from all patients in the dataset. In order to validate its application for patient risk stratification in the case of dengue it is necessary to properly understand how the features, phenotypes and categories behave in this newly reduced 2D space.

#### Analysis of features

**Figure 4:**
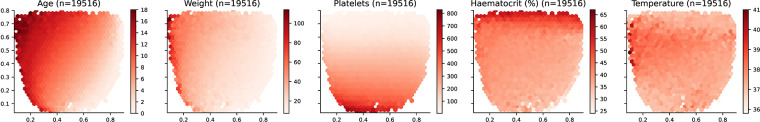
Latent space description: Features. The graphs represent the density distribution using hexagonal binning over the latent space for the five features selected to train the Autoencoder. The title includes the name and the number of daily profiles. The value on each hexagonal bin represents the mean value of all the daily profiles that have been projected on that bin. A detailed set of graphs describing other laboratory results has been included in Figure A1 (Appendix).

The distribution of the input features over the selected latent space is presented in Figure 4. Age and weight monotonically increase from the bottom-right corner towards the top-left corner. Note that this similitude is sensible since older patients, and more specifically in the case of children, tend to weigh more than younger patients. For platelet count, values monotonically increase from top towards bottom. On the contrary, haematocrit values increase from bottom towards top. It is important to highlight that body temperature does not present a monotonic increase and there is a horizontal region in the latent space around the y-axis value of 0.6 in which values are the highest. Further laboratory results are included in Figure A1 (Appendix).

#### Analysis of phenotypes

**Figure 5:**
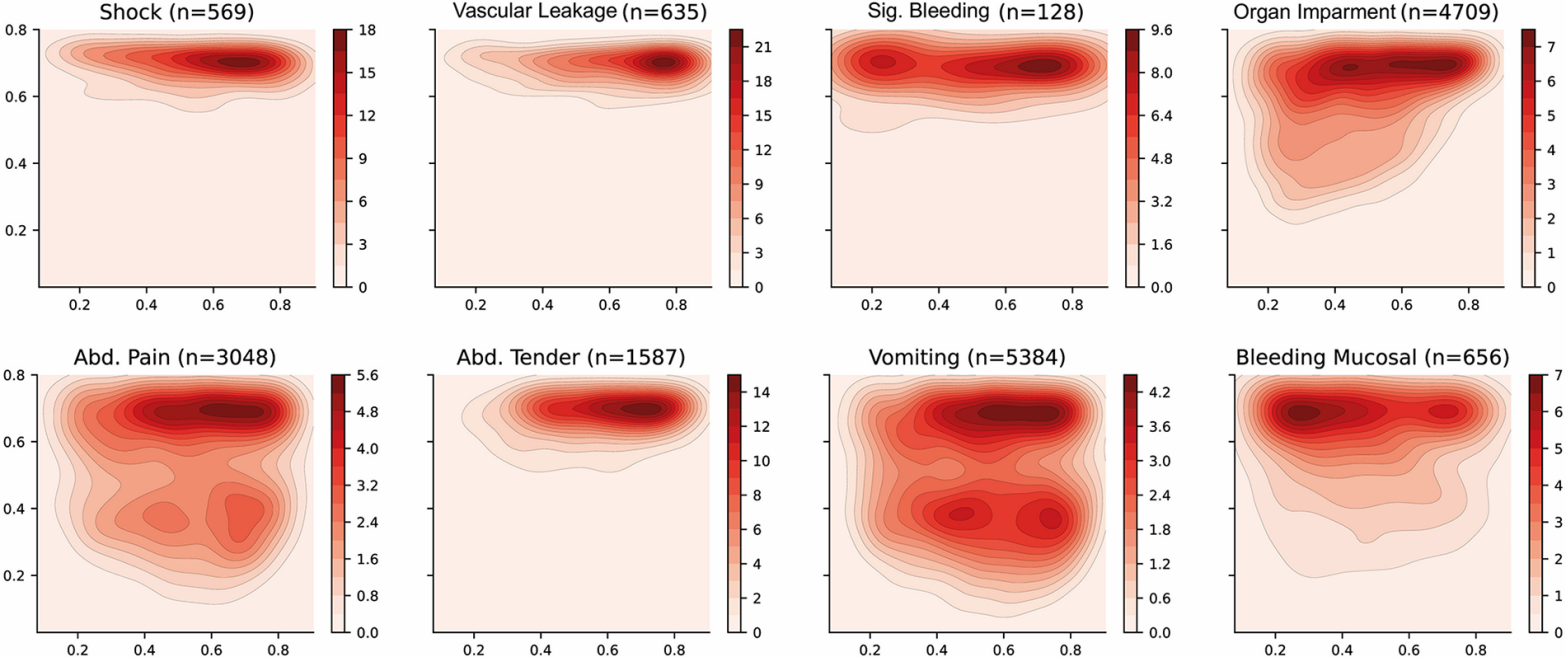
Latent space description: Phenotypes. The graphs represent the density distribution using contour lines estimated using a Gaussian kernel over the latent space. The title includes the phenotype and the number of patients in which it occurs. The definition of the compound categories (vascular leakage, significant bleeding and organ impairment) are defined in Table 2. A detailed set of graphs describing other phenotypes has been included in Figure A2 (Appendix).

The distribution of some interesting phenotypes of patients over the selected latent space is presented in Figure 5. Firstly, phenotypes describing similar conditions are shown to cover similar regions of the latent space—such as leakage related phenotypes (ascites, pleural effusion and pulmonary oedema) or bleeding-related phenotypes (nose bleeding, gum bleeding and other mucosal bleeding). Note that, in contrast to other bleeding sources, skin bleeding occurs more commonly in younger patients. The compound phenotypes for vascular leakage, significant bleeding, and organ impairment also occupy a very similar area, with leakage showing the highest density whereas organ impairment covers a larger area. This is reasonable since the latter is a broader classification as it includes abnormalities in the central nervous system (CNS), liver or kidney.

Dengue shock syndrome results in circulatory collapse (haemodynamic shock) and commonly occurs alongside abdominal pain and haemorrhage (bleeding). The corresponding density distributions are aligned on the right side of the latent space, which represents younger patients. There is also a clear bias towards collecting the features that have been defined as warning signs by the WHO dengue guidelines resulting in better estimated densities. For instance, vomiting and abdominal pain cover most of the latent space and are present in two areas with higher densities.

#### Analysis of categories

**Figure 6:**
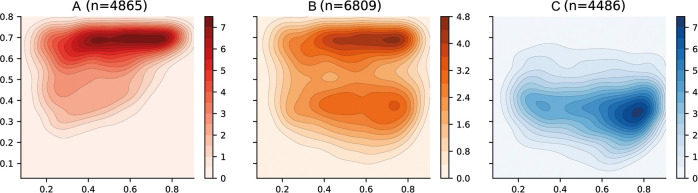
Latent space description: Categories. The graphs represent the density distribution over the latent space for three categories (A, B and C) estimated using a Gaussian kernel. These categories are defined as a compendium of various phenotypes (see Table 2) and therefore the value on each bin (pixel) described in the colorbar represents the estimated density on that bin for which one or more of the conditions associated to the category occurs.

The categories for patient risk stratification have been represented using density distributions in the latent space in Figure 6. Firstly, the *Category A* has been defined using those patients in which vascular leakage, significant bleeding, organ impairment or shock occurs (see Table 2). This region is also associated with those areas in which platelet levels are low and haematocrit levels are high (see Figure 4) which have been previously associated with severe dengue (33, 34). *Category B* has been defined using those patients in which any of the warning signs defined by WHO in the 2009 dengue guidelines (33) occur. There are two well differentiated regions with higher densities; the top region which overlies the *Category A* and the bottom region which overlies the *Category C*. Finally, the *Category C* category is defined by those patients who did not suffer any complication associated with either *Categories A* or *B*. This region is also associated with those areas in which platelet, haematocrit and body temperature values lie within their corresponding normal reference ranges. The region of the space with the higher density is shifted towards the right-hand side of the graph because the median age of patients in the overall data is 8 (see Table 6) and age increases from bottom-right to top-left (see age in Figure 4).

The course of the dengue infection is divided into three phases: febrile, critical, and recovery. The febrile phase is commonly associated with fever and, in some patients, the disease proceeds to a critical phase where fever resolves and an increase in haematocrit and a decrease in platelet levels are seen (34). This progression is clearly reflected in the latent space where in order to transition from *Category C* to *Category A* the patient tends to traverse a region in which body temperature values are the highest (see Temperature in Figure 4). This region of the latent space is therefore associated with the febrile phase.

#### Patient trajectories

**Figure 7:**
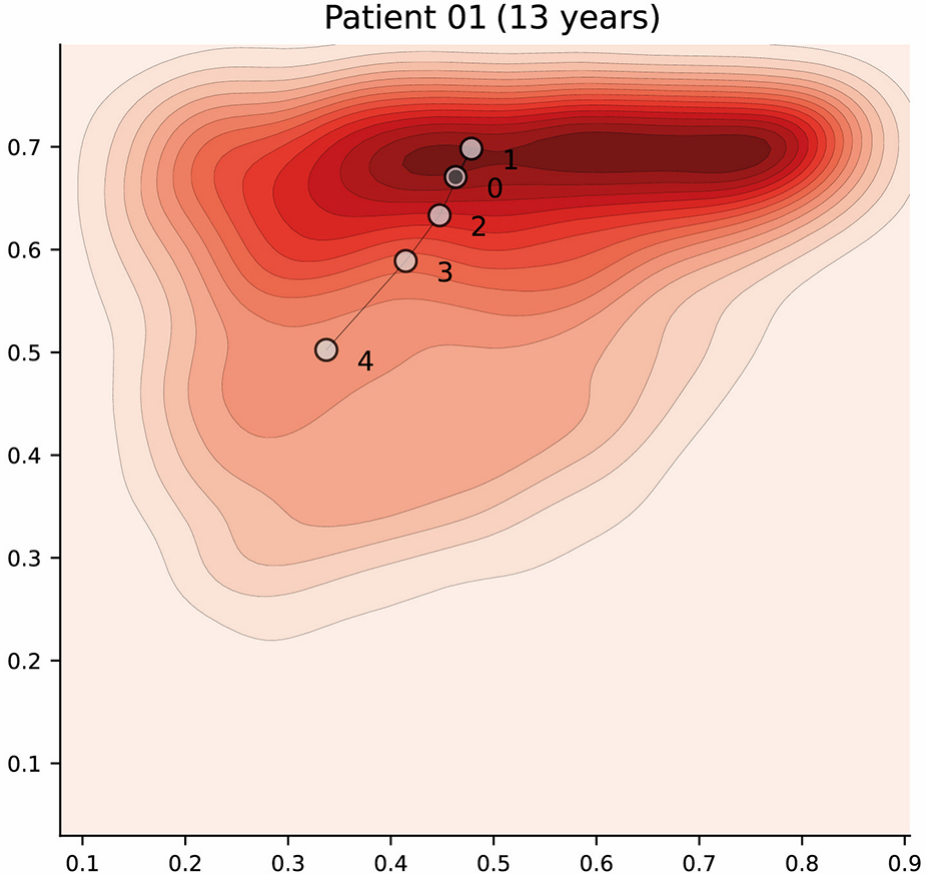
Latent space description: Trajectories. The graph represents the trajectory of a patient over the latent space using the density distribution for *Category A*, which associated with severe disease, as a background reference. Each marker represents a daily profile where the number indicates the day from admission. Filled markers indicate days in which the patient suffered an episode of shock. Further examples have been included in Figure A3 (Appendix).

Dengue is a dynamic acute illness and although an accurate snapshot is important, defining the changes over time is necessary. The selected autoencoder configuration preserves distances, therefore the latent space can also be used to visualise patient trajectories; that is, their evolution over time. Note that we could have more apparent clusters by increasing the complexity of the model, but it would affect the preservation of distances, and limit its usability for this particular scenario. An example of a patient trajectory is shown in Figure 7 where each marker represents a daily profile and the number indicates the day from admission. The filled markers indicate that the patient suffered an episode of shock on that day. The pattern that emerges in most patient trajectories is consistent; they are usually in the region associated with severe disease on admission to hospital (especially if they were admitted with shock) and move towards the mild region as they improve with treatment. Additionally, it is important to note that this is a descriptive method and should be used as such; assumptions regarding predicting patient states in the future are not made.

## Discussion

This study provides a proof-of-principle for the role of autoencoders to reduce the dimensionality of complex healthcare data into two dimensions to produce a latent space whereby regions are associated with specific clinical phenotypes. This descriptive tool provides visualisations of the latent space which subsequently allows for an intuitive and meaningful understanding of complex relationships between features for a given dataset. The clinical domain selected as an exemplar for this study was management of acute febrile illness including dengue, and the data an aggregation of various prospective clinical studies conducted between 1999–2021. The inclusion of over 12000 patients admitted to hospital represents the largest sample size used for this purpose to our knowledge.

The features used include routine clinical and laboratory parameters, selected based on expert consensus, data completeness and pragmatic utility suited to a LMIC healthcare setting. Although we have applied this technique to an infection condition using a limited set of clinical input parameters, this technique will likely be useful for a multitude of clinical conditions whereby the interplay between complex interacting patient and disease features need to be understood and classified to allow for clinical decision-making.

For the development of the method, it is necessary to clearly define the main objective of the model and chose adequate evaluation metrics. For similarity retrieval, distance metrics such as Pearson (distance preservation) or Spearman (rank preservation) should be used, whereas for visualisation density metrics are more suitable. For our purposes, which is a combination of both similarity retrieval and visualisation, balancing distance metrics and density metrics produces results covering most of the latent space, improving visualisation whilst preserving utility, with similar patients grouped closer together. In this case, balance is achieved by using the sigmoid activation function and one hidden layer with three neurons (Pearson, 0.840; Spearman, 0.830; Procrustes, 0.301; GMM 0.321). The latent space produced by the selected autoencoder aligns with established characteristics of dengue disease progression, such as an increase in haematocrit levels, decrease in platelet levels and a decrease in body temperature from febrile to critical phase (33, 34). The same occurs for other laboratory tests not used during training which are often indicators of disease severity such as high AST, high ALT, respiratory distress (respiratory rate), high pulse and low pulse pressure (see Figure A1 in Appendix). Moreover, similar phenotypes are represented close to each other, and the categories defined are consistent with both the magnitude features (e.g. abdominal pain magnitude) which are subject to clinicians interpretation and the clinical warning signs outlined in the WHO 2009 dengue guidelines (33).

### Benefits

In supervised learning the objective is to learn a function that maps an input (features) to an output (phenotypes) based on input-output pairs. This imposes the need to define a standardised output label for which the model is optimised. The models produced are therefore more prone to overfit and predictions are constrained to the selected output. The use of unsupervised learning by means of autoencoders overcomes these limitations as it finds patterns within the data based exclusively on the input. The latent space produced can be easily described without the need for model retraining. In this manuscript, we have described the latent space in terms of features and phenotypes. Moreover, compound features and even categories can be defined “online” without retraining which provides immense flexibility. In addition, the visualisation aspect considered during the development of the model and the thorough description of the latent space improves understanding and confidence - with the ultimate aim of promoting a superior adoption among clinicians in comparison with conventional approaches. The understanding of the relationships between patients through the input features when reduced to a latent space representation could also offer additional insights and understanding of relationships hitherto not explicitly defined – this is useful for research particularly when complemented with additional investigations such as through biomarker and genomic methodologies.

### Limitations

Since there is no target or outcome variable, unsupervised learning is more technically challenging than supervised learning and requires more input from subject-matter experts. This is true especially for feature selection and validation of the produced latent space. In addition, as with other approaches that rely on a voting system, predictions are not recommended if the phenotype of interest is highly imbalanced. The nature of this technique is to depict representations of data relationships and not to provide inference/predictions of future patient states. Note however that there are other strategies that can be used to raise alerts. For instance, by assessing whether the density (number of patients) for which a certain phenotype occurs is considerably higher in the region surrounding the query patient compared to the rest of the space. There were limitations on the dataset used—patient data was collected on the basis of enrolment to a clinical study and therefore subject to a selection bias according to the inclusion criteria, and clinical information for patients not diagnosed with dengue were relatively sparse compared with those with laboratory-confirmed dengue. The input features (5 parameters) were also relatively limited - additional features collected longitudinally would likely be able to provide increased specificity and performance. However, we have shown that despite the use of minimal features, meaningful representations of outcome categories in dengue can be constructed and displayed.

## Conclusion

Autoencoders, when adequately configured, can produce a two-dimensional latent space representation of a complex dataset of dengue patients collected over 20 years which (i) conserves the distance and rank relationships between patients, (ii) aligns with important clinical characteristics in patients with dengue and (iii) groups patients with similar phenotypes close together. The data used in this study was collected manually by clinicians and the features have been selected to maximize the number of patients based on expert consensus and data availability. However, one of the strengths of this method is that it has the potential to perform well with higher dimensional data including time-series or even images. The parametric model produced during training in the form of weights and biases can be used to encode new, previously unseen data points and thus represent these patients in the latent space. The encoding is done in constant time, making a real-time patient similarity retrieval system possible. Work is underway to evaluate its utility in facilitating end user data interpretation by incorporating these findings into an electronic clinical decision support system to guide individual patient management.

## Figures and Tables

**Appendix A1:**
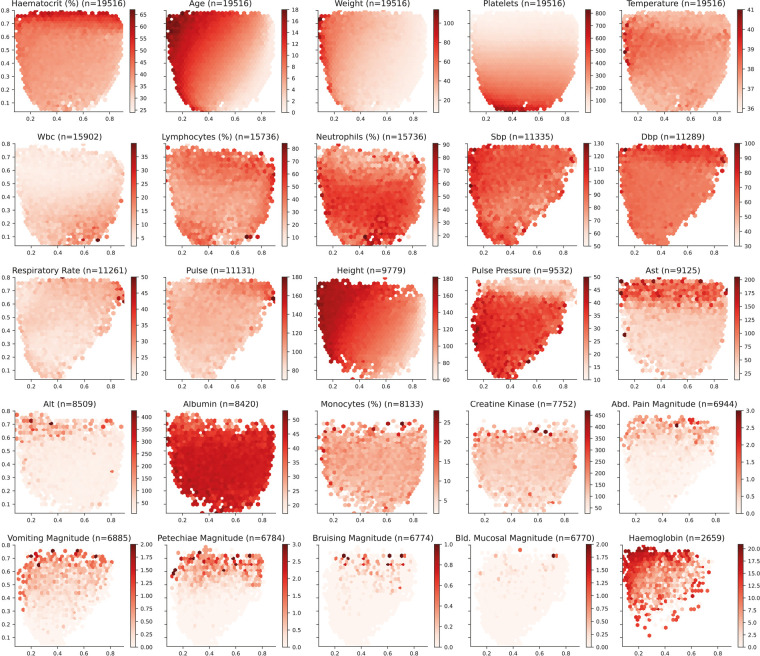
Latent space description: Biomarkers. The graphs represent the density distribution using hexagonal binning over the latent space for the most frequent features in the dataset. The title includes the name and the number of daily profiles. The value on each hexagonal bin represents the mean value of all the daily profiles that have been projected on that bin. The magnitude values are subject to the clinicians interpretation and have been included for reference.

**Appendix A2:**
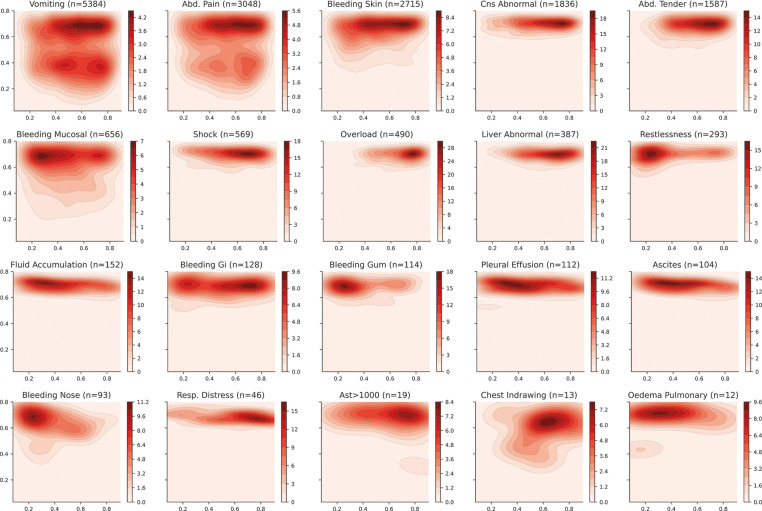
Latent space description: Phenotypes. The graphs represent the density distribution using contour lines estimated using a Gaussian kernel over the latent space. The title includes the phenotype and the number of patients in which it occurs.

**Appendix A3:**
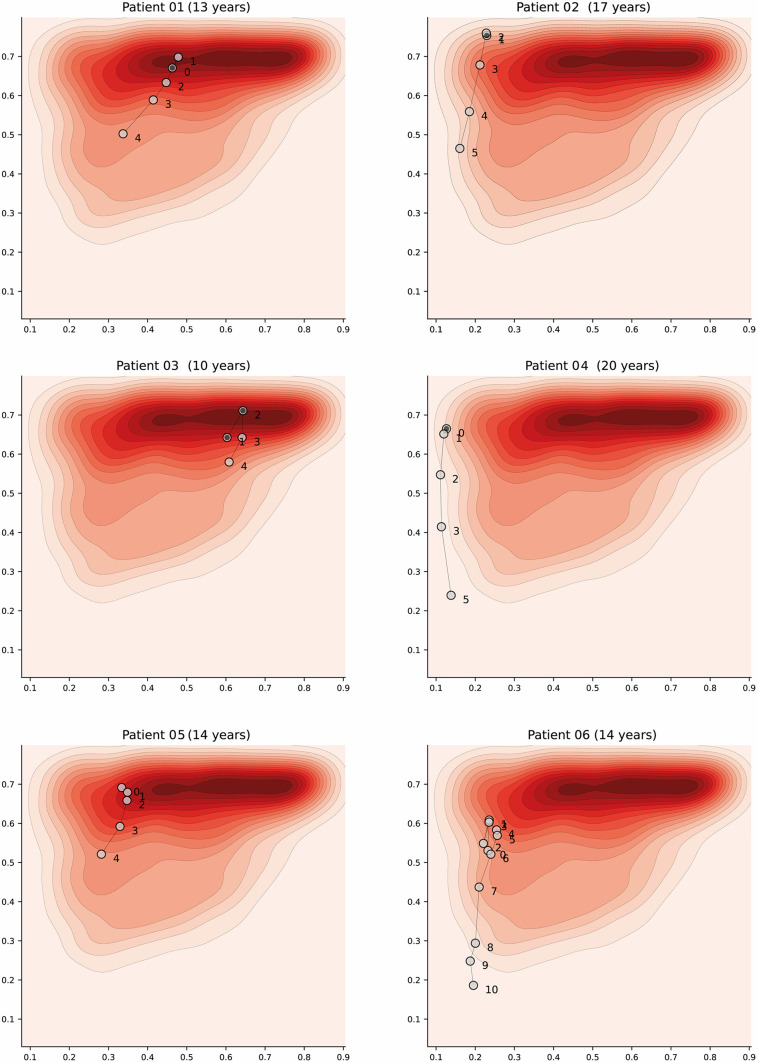
Latent space description: Trajectories. The graphs represent trajectory of patients over the latent space using the density distribution for the Category A (which is associated with higher severity) as a background reference. Each marker represents a daily profile where the number indicates the day from admission. Filled markers indicate days in which the patient suffered an episode of shock.

## Data Availability

The anonymised datasets analysed during the current study are available from the corresponding author (BH b.hernandez-perez@imperial.ac.uk) on reasonable request, as long as this meets local ethics and research governance criteria. The code can be accessed at https://github.com/bahp/fdgth.2023.1057467.
