# Supplementary material for: Learning meaningful latent space representations for patient risk stratification: Model development and validation for dengue and other acute febrile illness
Source: Front Digit Health. 2023 Feb 22;5:1057467. doi: 10.3389/fdgth.2023.1057467 (PMC9992802; doi:10.3389/fdgth.2023.1057467)
Supplement: Supplementary file 1 [file Datasheet1.pdf]

Supplementary Appendix

Appendix I – Description of data source and individual studies 2
Appendix II – Visualisation of patients on the selected latent space 14
Appendix III – Dealing with missing data 15

Supplementary appendix I

Description of data source

Prospective clinical studies conducted in Vietnam by Oxford University Clinical Research Unit between 1999 and 2021 were used to derive the final dataset used for analyses. Electronic data from the following studies were accessed after a data sharing agreement between Imperial College London and Oxford University Clinical Research Unit (OUCRU) / Hospital for Tropical Diseases (HTD) in March 2020. A summary of the studies including the baseline characteristics of patients is shown in Table 1:

Table 1: Overview of clinical studies.

No.	Code	Recruitment dates	Number of patients (included/enrolled)	Title of study
S1[1,2,3]	DF	1999 – 2009	1,660/1,719	Inpatient-based prospective observational descriptive study of clinical features of DSS in children and comparison of different fluid solutions for initial resuscitation
S2[4]	MD	2001 – 2009	1,197/3,044	Inpatient-based prospective observational descriptive study examining prognostic factors during the febrile phase of non-severe dengue in children
S3[5]	DR	2005 – 2009	1,536/1,542	Outpatient-based prospective observational descriptive study to examine features of non-severe dengue in children
S4[6]	FL	2006 – 2008	190/738	An inpatient-based prospective descriptive study of patients with dengue
S5[7,8]	06DX	2009 – 2011	213/225	A pilot study to investigate the effects of short course oral corticosteroid therapy in early dengue infection in Vietnamese patients
S6[9,10,11]	13DX	2010 – 2014	8,016/8,100	Laboratory diagnosis and prognosis of severe dengue
S7[12,13]	32DX[t]	2013 – 2015	10/75	Inpatient-based prospective observational descriptive study to characterise microvascular and endothelial function
S8	42DX	2016 – 2018	16/664	A matched cohort study to characterise the clinical manifestations of dengue in pregnancy and investigate the spectrum of adverse maternal and fetal outcomes.
S9	01NVA	2020 – 2021	46/155[*]	Innovative biomedical engineering and computational science to improve the management of critical illness in resource-limited settings

[*] At the time of analysis. Further patients are being recruited.

[t] This was a multi-centre study conducted at the National Hospital for Tropical Diseases (NHTD), Hanoi and the Hospital for Tropical Diseases (HTD), Ho Chi Minh City, Vietnam with 75 and 103 patients recruited respectively. Only the former has been considered.

Details of individual studies

S1. "Inpatient-based prospective observational descriptive study of clinical features of DSS in children and comparison of different fluid solutions for initial resuscitation" (DF)

Recruitment details

This was a single centre, observational study of children with DSS. The study took place in the paediatric intensive care unit at the Hospital for Tropical Diseases (HTD) in Ho Chi Minh City, Vietnam. The first phase consisted of a randomized, double-blind comparison of an isotonic crystalloid solution (Ringer's lactate) and two isotonic colloid solutions (6% hydroxyethyl starch 200/0.5 and 6% dextran 70) for emergency resuscitation of children (aged 2 to 15 years) with DSS. WHO guidelines were used for the diagnosis of DSS. No children with profound shock (pulse pressure <=10 mm Hg at presentation with shock) received a crystalloid because of concerns about the potential development of critical fluid overload without access to advanced respiratory support.

After completion of the trial, the study continued in a similar format but with no randomization. During this phase, all study participants received a standardised regime of fluid therapy in line with HTD's DSS management guidelines. A total of 1,719 patients were recruited, of whom 512 participated in the fluid trial.

Dengue diagnosis

A diagnosis of dengue infection was made with the use of Dengue Duo IgM capture and IgG capture enzyme-linked immunosorbent assay kits (PanBio) on paired serum samples. Coagulation screening was performed with the use of kits obtained from Diagnostica Stago; tests included those for prothrombin time, activated partial-thromboplastin time, and fibrinogen level and a semiquantitative assay for fibrin-degradation products.

Outcomes

The fluid trial results showed that initial resuscitation with Ringer's lactate is indicated for children with moderately severe dengue shock syndrome. Dextran 70 and 6% hydroxyethyl starch performed similarly in children with severe shock, but given the adverse reactions associated with the use of dextran, starch may be preferable for this group. In addition, the trial showed that with prompt intervention and assiduous clinical care by experienced staff, the outcome of paediatric DSS can be excellent.

The larger dataset was used to explore risk factors for development of profound or recurrent shock among children presenting with DSS - age, day of illness, high pulse, high temperature, or high haematocrit levels were identified, and a prognostic model for development of profound shock was developed.

S2. Inpatient-based prospective observational descriptive study examining prognostic factors during the febrile phase of non-severe dengue in children (MD)

Recruitment details

This was a prospective inpatient observational study recruiting children between 5 and 15 years admitted to the paediatric dengue ward at the HTD, Ho Chi Minh City between 12/4/2001and 24/7/2009. A total of 3,042 patients were recruited to the study with confirmed dengue. Participants who were admitted to the paediatric ward at the HTD were those with uncomplicated illness only who were not suitable for outpatient care. Patients with severe dengue or require frequent (3-6 hourly) monitoring were admitted or transferred to the paediatric intensive care unit. A total of 3,044 children with suspected dengue were enrolled.

Dengue diagnosis

Diagnosis of dengue was confirmed by detection of dengue virus (DENV) RNA in plasma by reverse transcriptase polymerase chain reaction (RT-PCR) at enrolment, or by seroconversion on IgM and IgG capture ELISA on paired enrolment and early convalescent specimens (Dengue Duo IgM and IgG Capture ELISA, PanBio, Australia) or in-house methods.

Outcomes

The data were used to describe the spectrum of clinical manifestations among children hospitalised with dengue and to explore risk factors for progression to DSS, the most common manifestation of severe dengue in this population. This was defined as narrow pulse pressure (≤ 20 mmHg) or hypotension for age with evidence of impaired peripheral perfusion.

S3. Outpatient-based prospective observational descriptive study to examine features of non-severe dengue in children (DR)

Recruitment details

This was a prospective descriptive study of febrile children, aged 5-15 years, attending two primary health care clinics in Ho Chi Minh City, Vietnam. Clinic A is a single-handed practice run by a senior paediatrician, while Clinic B is the walk-in paediatric clinic at District 8 Hospital. All children presenting with fever and clinically suspected dengue to either clinic were eligible for enrolment. Recruitment was targeted towards patients presenting during the early febrile period, ideally within the first 72h from fever onset, although patients presenting up to 96h from fever onset could be enrolled. A total of 1,542 patients were enrolled.

Dengue diagnosis

Diagnosis of dengue was confirmed by detection of dengue virus (DENV) RNA in plasma by reverse transcriptase polymerase chain reaction (RT-PCR) at enrolment, or by seroconversion on IgM and IgG capture ELISA on paired enrolment and early convalescent specimens (Dengue Duo IgM and IgG Capture ELISA, PanBio, Australia) or in-house methods[6].

Outcomes

The description of the characteristics of dengue disease in children presenting to outpatient clinical facilities, and assessment of risk factors for hospitalisation and/or progression to severe disease were the main endpoint of this study. In addition, the assessment of microalbuminuria for early diagnosis and risk prediction constituted a secondary endpoint.

S4. “Inpatient-based prospective descriptive study of patients with dengue” (FL)

Recruitment details

This was a study that prospectively collected information across the full spectrum of hospitalised dengue cases admitted to a single hospital in southern Vietnam to compare clinical and laboratory features, management, and outcome in 647 adults and 881 children with confirmed dengue. All patients recruited on the ICUs during the two years between September 2006 and September 2008 and all patients recruited in the infection wards during the year 2007. The following groups are eligible for recruitment after appropriate written informed consent: a) children 2–15 years old admitted to the Paediatric Intensive Care Unit (PICU) with clinically suspected dengue and overt complications; b) patients ≥15 years old with suspected dengue admitted to the Adult Intensive Care Unit (AICU) for any reason; c) children 5–15 years old or adults ≥15 years old admitted to the paediatric or adult infection wards with suspected dengue. Eligibility criteria were deliberately broad to allow the study physicians to invite all potential subjects with suspected dengue to participate.

Dengue diagnosis

Dengue diagnostic capture IgM and IgG ELISA assays were performed using paired enrolment and convalescent specimens and reagents provided by Venture Technologies (Sarawak, Malaysia). Using the enrolment specimen, DENV RT-PCR was carried out for all paediatric patients (who were generally admitted early) and all adult patients enrolled within the first 5 days of illness, as well as those with negative or inconclusive serology. If both serology and RT-PCR were negative or inconclusive, assays for non-structural protein 1 (NS1) were performed using Biorad Platelia™ Dengue NS1 Antigen kits following the manufacturer's instructions. Patients were diagnosed to have confirmed dengue if the RT-PCR and/or NS1 results were positive, seroconversion was documented on paired serology, or dengue specific IgM was identified in a patient with a typical clinical syndrome. To define immune status the ratio of IgM to IgG on or after day 6 of illness was used; a ratio ≥ 1.78 defined the infection as primary and one ≤1.2 as secondary. Patients with ratios between these values, or in whom the results of enrolment and convalescent specimens differed, were considered unclassifiable.

Outcomes

The main analysis identified clear distinctions between adults and children in the pattern of complications seen in association with dengue infection and indicated that these depend partly on intrinsic age-dependent physiological differences.

S5. “A pilot study to investigate the effects of short course oral corticosteroid therapy in early dengue infection in Vietnamese patients” (06DX)

Recruitment details

This was a randomised placebo-controlled clinical trial examining the safety and efficacy of low or high dose oral prednisolone in preventing disease progression, among patients aged 5-20 years admitted to the HTD. In total, 225 participants, all with confirmed dengue, were enrolled – these patients had a fever onset within 72 hours and had no signs of dengue-related complications at enrolment, and no significant past medical history or need for regular medication. Among the 225 trial participants, 75 patients were included in each treatment group.

Dengue diagnosis

Diagnosis was supported through a positive rapid test for dengue non-structural protein 1 (NS1 Ag-STRIP, Bio-Rad) and confirmed through either a DENV RT-PCR at enrolment or a capture ELISA IgM and IgM using paired specimens (Venture Technologies, Sarawak, Malaysia) within 72 hours of illness onset and at day 7.

Outcomes

Use of oral prednisolone during the early acute phase of dengue infection was not associated with prolongation of viremia or other adverse effects. Although not powered to assess efficacy, no reduction in development of shock or other recognized complications of dengue virus infection was apparent in this study.

S6. “Laboratory diagnosis and prognosis of severe dengue” (13DX)

Recruitment details

This was a prospective community-based observational study recruiting patients presenting to outpatient departments at seven large hospitals in Southern Vietnam – these include the HTD, Children’s Hospital 1 and 2 in Ho Chi Minh city, Dong Nai children hospital, Long An provincial hospital, Tien Giang hospital, and Binh Duong hospital. Children between 1-15 years of age attending the outpatient department with a fever 3 days or less in whom dengue was a possible diagnosis were eligible for enrolment. The exclusion criteria included cases where an alternative diagnosis was considered to be more likely or if the participant would be unlikely to be able to attend follow up. In total 8,100 participants were enrolled in the study of whom 2,245 had confirmed dengue and 940 patients who were hospitalised were included in the analyses.

Diagnosis

A diagnosis of dengue was confirmed through one of the following: positive RT- PCR for DENV, a positive NS1 assay (Platelia NS1 antigen or NS1 Ag STRIP, Bio-Rad, France) at enrolment or detection of IgM seroconversion.

Outcomes

Dengue shock syndrome, dengue with severe bleeding, or dengue with end-organ involvement (central nervous dysfunction, hepatic dysfunction or severe respiratory dysfunction or other major organ involvement) constituted the main study endpoints. The study demonstrated a) that early diagnosis of dengue can be enhanced beyond the current standard of care using a simple evidence-based algorithm and b) that use of a simple prognostic model (the Early Severe Dengue Identifier) that included history of vomiting, platelet count, AST level and NS1 rapid test status performed reasonably well in predicting development of severe dengue during the early febrile phase.

S7. “Inpatient-based prospective observational descriptive study to characterise microvascular and endothelial function.” (32DX)

Recruitment details

This was a prospective observational study conducted at the Hospital for Tropical Diseases (HTD), Ho Chi Minh City and the National Hospital for Tropical Diseases (NHTD), Hanoi, Vietnam, between June 2013 and October 2015. Patients with dengue admitted to either paediatric or adult ICU were enrolled within 12 hours of admission. All patients were reviewed daily until hospital discharge or for up to 5 days from enrolment; at each assessment standardized clinical information was recorded including clinical symptoms and signs, vital signs and all interventions. The amount and type of all intravenous fluids were documented. Portable echocardiograms were performed as soon as feasible after enrolment, and then daily until discharge from ICU. The patients were followed up 10–14 days later.

Diagnosis

Commercial IgM and IgG serology assays (Capture ELISA, Panbio, Australia) were performed on batched acute and convalescent plasma. In addition, RT-PCR was performed on the enrolment sample to identify the DENV serotype and measure plasma viremia levels. Patients were defined as having dengue if the RT-PCR was positive or if the IgM assays were positive at enrolment, or IgM seroconversion between paired specimens and on the basis of their clinical picture. Patients with negative tests at enrolment, but for whom convalescent plasma was not available, were considered unclassifiable.

Outcomes

Echo-derived intravascular volume assessment and venous lactate levels can help identify dengue patients at high risk of recurrent shock and respiratory distress in ICU. Endothelial dysfunction/NO bioavailability is associated with worse plasma leakage, occurs early in dengue illness, and correlates with hypoargininemia and high arginase-1 levels.

S8. "A matched cohort study to characterise the clinical manifestations of dengue in pregnancy and investigate the spectrum of adverse maternal and fetal outcomes." (42DX) – unpublished data

Recruitment details

This was a prospective observational study recruiting women with acute dengue during pregnancy matched with non-pregnant female controls with acute dengue at the HTD in Ho Chi Minh City. Women between 18 and 45 years old referred to the HTD with a fever and clinical symptoms consistent with dengue and who were either pregnant or not during the recruitment period were eligible. The matching criteria were patients' age and day of illness at enrolment. In total 664 patients with suspected dengue (82% subsequently confirmed, of whom 212 were pregnant and 327 were not)) were recruited.

Diagnosis

Diagnosis of dengue was confirmed by detection of DENV-RNA in plasma by RT-PCR, NS1 antigen detection at enrolment or by IgM seroconversion using conventional serological methods.

Outcomes

The data are being used to provide a comprehensive description of differences and similarities in clinical dengue disease and risk for complications between pregnant and not pregnant women, and to explore potential effects of dengue during pregnancy on fetal growth and neurodevelopment up to 2 years of age.

S9. “Physiological waveform analysis to predict severity of disease in dengue and sepsis” (01NVA) – unpublished data

Recruitment details
This is a prospective observational study recruiting patients with dengue at the HTD in Ho Chi Minh City. Patients aged 8 years or above referred to the HTD with a clinical diagnosis of dengue are eligible, aiming for recruitment within 48 hours from clinical diagnosis at hospital admission. The study started in March 2020 and is still recruiting with the overall target of 250. On enrolment patients’ baseline clinical information is recorded, then continuous physiological data recording takes place over a 24-hour period. All patients are reviewed daily until hospital discharge or for up to 5 days from enrolment; at each assessment standardised clinical information is recorded including clinical symptoms and signs, vital signs and all intervention. There is no follow-up visit in this study.

Diagnosis
The definitions for clinical diagnosis have been selected as these are the commonly used definitions at HTD and therefore represent the ‘real life’ clinical situation at HTD; however these clinical definitions are based on the WHO 2009 dengue classification system. Diagnosis is also supported through a positive rapid test for dengue non-structural protein 1 (NS1) and the detection of DENV IgM at discharge if done.

Outcomes
The primary outcome is the ability to evaluate fluid status and predict shock/reshock and organ failure. The secondary outcomes are development and validation of machine learning algorithms to compare the concordance of PPG pulse waveform data with clinical measurements, namely blood pressure, respiratory rate and haematocrit measured routinely as standard of care during admission.

References

1. Wills BA, Nguyen MD, Ha TL, Dong TH, Tran TN, Le TT, et al. Comparison of three fluid solutions for resuscitation in dengue shock syndrome. N Engl J Med. 2005;353(9):877-89. https://pubmed.ncbi.nlm.nih.gov/16135832/

2. Lam PK, Tam DTH, Diet TV, Tam CT, Tien NTH, Kieu NTT, et al. Clinical Characteristics of Dengue Shock Syndrome in Vietnamese Children: A 10-Year Prospective Study in a Single Hospital. Clin Infect Dis. 2013;57(11):1577-86.
https://pubmed.ncbi.nlm.nih.gov/24046311/

3. Lam PK, Tam DT, Dung NM, et al. A Prognostic Model for Development of Profound Shock among Children Presenting with Dengue Shock Syndrome. PLoS One. 2015;10(5):e0126134. Published 2015 May 6. doi:10.1371/journal.pone.0126134
https://pubmed.ncbi.nlm.nih.gov/25946113/

Lam PK, Ngoc TV, Thu Thuy TT, Hong Van NT, Nhu Thuy TT, Hoai Tam DT, et al. The value of daily platelet 4. counts for predicting dengue shock syndrome: Results from a prospective observational study of 2301 Vietnamese children with dengue. PLoS Negl Trop Dis. 2017;11(4):e0005498.
https://pubmed.ncbi.nlm.nih.gov/28448490/

5. Hanh Tien NT, Lam PK, Duyen HT, et al. Assessment of microalbuminuria for early diagnosis and risk prediction in dengue infections. PLoS One. 2013;8(1):e54538. doi:10.1371/journal.pone.0054538
https://journals.plos.org/plosone/article?id=10.1371/journal.pone.0054538

6. Dinh The T, Le Thi Thu T, Nguyen Minh D, Tran Van N, Tran Tinh H, Nguyen Van Vinh C, et al. Clinical Features of Dengue in a Large Vietnamese Cohort: Intrinsically Lower Platelet Counts and Greater Risk for Bleeding in Adults than Children. PLoS Negl Trop Dis. 2012;6(6):e1679.
https://www.ncbi.nlm.nih.gov/pmc/articles/PMC3383761/

7. Tam DTH, Ngoc TV, Tien NTH, Kieu NTT, Thuy TTT, Thanh LTC, et al. Effects of Short-Course Oral Corticosteroid Therapy in Early Dengue Infection in Vietnamese Patients: A Randomized, Placebo-Controlled Trial. Clin Infect Dis. 2012;55(9):1216-24.
https://pubmed.ncbi.nlm.nih.gov/22865871/

8. Nguyen, T. H., Nguyen, T. H., Vu, T. T., Farrar, J., Hoang, T. L., Dong, T. H., Ngoc Tran, V., Phung, K. L., Wolbers, M., Whitehead, S. S., Hibberd, M. L., Wills, B., & Simmons, C. P. (2013). Corticosteroids for dengue - why don't they work? PLoS Negl Trop Dis, 7(12), e2592. https://doi.org/10.1371/journal.pntd.0002592
https://www.ncbi.nlm.nih.gov/pmc/articles/PMC3861232/

9. Nguyen MT, Ho TN, Nguyen VV, et al. An Evidence-Based Algorithm for Early Prognosis of Severe Dengue in the Outpatient Setting. Clin Infect Dis. 2017;64(5):656-663. doi:10.1093/cid/ciw863
https://pubmed.ncbi.nlm.nih.gov/28034883/

10. Tuan NM, Nhan HT, Chau NV, et al. Sensitivity and specificity of a novel classifier for the early diagnosis of dengue. PLoS Negl Trop Dis. 2015;9(4):e0003638. Published 2015 Apr 2. doi:10.1371/journal.pntd.0003638
https://pubmed.ncbi.nlm.nih.gov/25836753/?from_term=tuan+dengue&from_pos=3

11. Yacoub S, Wertheim H, Simmons CP, Screaton G, Wills B. Microvascular and endothelial function for risk prediction in dengue: an observational study. Lancet. 2015;385 Suppl 1:S102.
https://pubmed.ncbi.nlm.nih.gov/26312832/

12. Yacoub S, Hunh T, Kanh LP, Broyd C, Quyen NTH, Duyen HTL, Simmons CP, Screaton G, Wills B. Cardio-haemodynamic assessment and venous lactate in severe dengue: relationship with recurrent shock and respiratory distress. Plos Negl Trop Dis 2017 Jul 10;11(7)
https://pubmed.ncbi.nlm.nih.gov/28692675/

13. Yacoub S, Hunh T, Kanh LP, Quyen NTH, Duyen HTL, Mongkolsapaya J, Culshaw A, Wertheim H, Simmons CP, Screaton G, Wills B. Endothelial nitric oxide pathways in the pathophysiology of dengue disease

progression: an observational study. Clin Infect Dis 2017 Jun 29.
https://www.ncbi.nlm.nih.gov/pmc/articles/PMC5850435/

Supplementary appendix II

Visualisation of patients on the selected latent space

Prospective clinical studies conducted in Vietnam by Oxford University Clinical Research Unit between 1999 and 2021 were used to derive the final dataset used for analyses. The characteristics of the patients recruited on each study considerably different and such variability must be retained on the latent space to produce meaningful embeddings suitable for similarity retrieval. Figure 1 shows the embeddings of the patients by clinical study and demonstrates that the selected autoencoder is suitable for similarity retrieval.

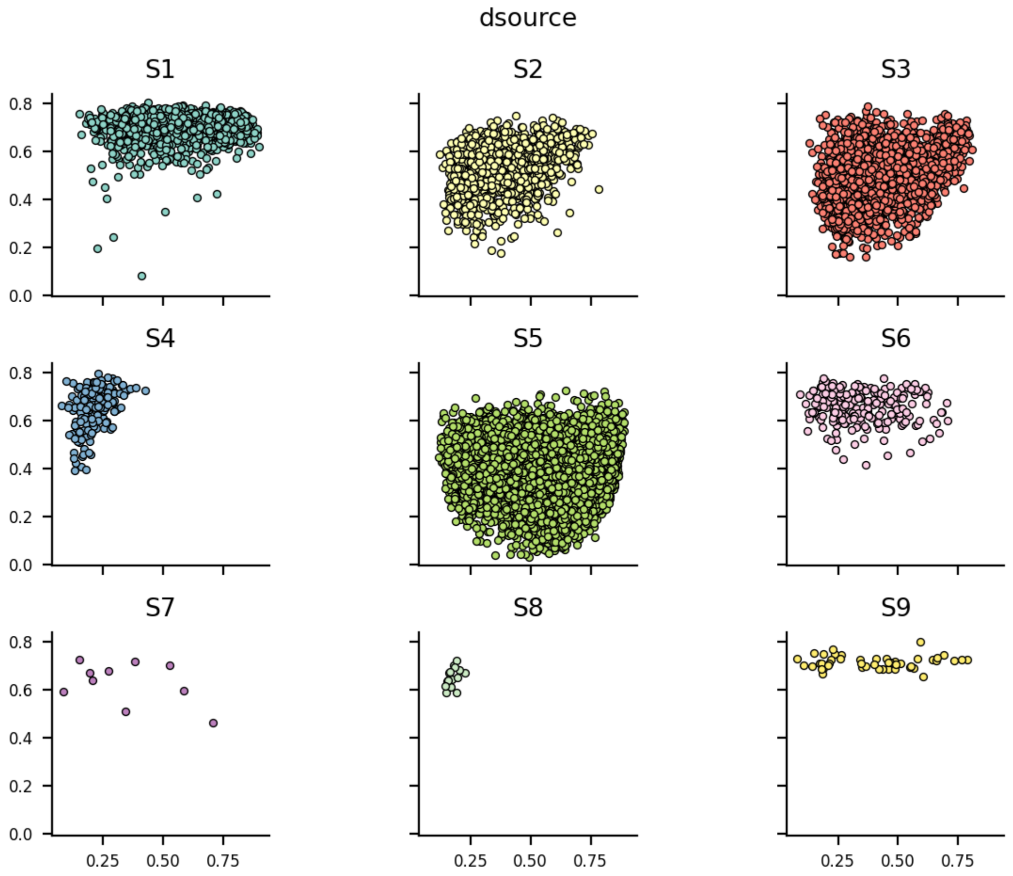

Figure 1. Visualisation of patients' embeddings by clinical study

Supplementary appendix III

Dealing with missing data

This study has been designed carefully to include a minimal number of routinely available features that are well-known to be associated with dengue disease severity and progression. The aim is to reduce complexity of data collection, improve understanding within users (clinicians) and therefore facilitate both deployment an adoption of the system in real-world settings with specific focus on low- and middle-income countries. Moreover, complexity is also often tightly linked with increased costs. For instance, the feature set selected as input to the model might be constrained by the laboratory testing and financial capabilities of the institution.

For scenarios in which missing data is available, the imputation methodology varies considerably depending on the problem domain and the feature itself. In research, two main approaches are used with their own benefits and drawbacks: (i) simple imputation (e.g. using the population mean or mode) or (i) inference based using the available features. The former is quite simple and often assumes that the reason for the variable not being available is because it is not relevant; that is, imputing the mean reflects the majority (e.g. healthy patients). However, if this assumption doesn't hold the method will introduce bias. The inference-based approach might be a better option if the available features are shown to be feasible predictors, but this needs to be validated. Overall, these two techniques tend to lead to over-inflated performance during retrospective evaluation since the ML algorithm is often able to identify simple imputation patterns. Another quite convenient approach is to replicate the way it is done in clinical practice. For instance, for temporal data such as time series, using the last available value seems reasonable since it replicates the way it is done during practice.
